# Complete genome sequences of two strains of *Treponema pallidum* subsp. *pertenue* from Indonesia: Modular structure of several treponemal genes

**DOI:** 10.1371/journal.pntd.0006867

**Published:** 2018-10-10

**Authors:** Michal Strouhal, Lenka Mikalová, Jan Haviernik, Sascha Knauf, Sylvia Bruisten, Gerda T. Noordhoek, Jan Oppelt, Darina Čejková, David Šmajs

**Affiliations:** 1 Department of Biology, Faculty of Medicine, Masaryk University, Brno, Czech Republic; 2 Work Group Neglected Tropical Diseases, Infection Biology Unit, German Primate Center, Leibniz Institute for Primate Research, Goettingen, Germany; 3 Public Health Laboratory, Department of Infectious Diseases GGD Amsterdam, WT Amsterdam, the Netherlands; 4 Izore, Centrum Infectieziekten Friesland, EN Leeuwarden, the Netherlands; 5 CEITEC-Central European Institute of Technology, Masaryk University, Brno, Czech Republic; 6 National Centre for Biomolecular Research, Faculty of Science, Masaryk University, Brno, Czech Republic; 7 Department of Immunology, Veterinary Research Institute, Brno, Czech Republic; University of Connecticut Health Center, UNITED STATES

## Abstract

**Background:**

*Treponema pallidum* subsp. *pertenue* (TPE) is the causative agent of yaws, a multistage disease endemic in tropical regions in Africa, Asia, Oceania, and South America. To date, seven TPE strains have been completely sequenced and analyzed including five TPE strains of human origin (CDC-2, CDC 2575, Gauthier, Ghana-051, and Samoa D) and two TPE strains isolated from the baboons (Fribourg-Blanc and LMNP-1). This study revealed the complete genome sequences of two TPE strains, Kampung Dalan K363 and Sei Geringging K403, isolated in 1990 from villages in the Pariaman region of Sumatra, Indonesia and compared these genome sequences with other known TPE genomes.

**Methodology/principal findings:**

The genomes were determined using the pooled segment genome sequencing method combined with the Illumina sequencing platform resulting in an average coverage depth of 1,021x and 644x for the TPE Kampung Dalan K363 and TPE Sei Geringging K403 genomes, respectively. Both Indonesian TPE strains were genetically related to each other and were more distantly related to other, previously characterized TPE strains. The modular character of several genes, including TP0136 and TP0858 gene orthologs, was identified by analysis of the corresponding sequences. To systematically detect genes potentially having a modular genetic structure, we performed a whole genome analysis-of-occurrence of direct or inverted repeats of 17 or more nucleotides in length. Besides in *tpr* genes, a frequent presence of repeats was found in the genetic regions spanning TP0126–TP0136, TP0856–TP0858, and TP0896 genes.

**Conclusions/significance:**

Comparisons of genome sequences of TPE Kampung Dalan K363 and Sei Geringging K403 with other TPE strains revealed a modular structure of several genomic loci including the TP0136, TP0856, and TP0858 genes. Diversification of TPE genomes appears to be facilitated by intra-strain genome recombination events.

## Author summary

*Treponema pallidum* subsp. *pertenue* (TPE) is the causative agent of yaws, a multi-stage disease that is endemic in tropical regions of Africa, Asia, Oceania, and South America. TPE belongs to the pathogenic treponemes and causes several human and animal infections. Whole genome sequences of two TPE strains isolated from patients in Indonesia were determined in this study. While both strains were highly related to other TPE strains isolated from humans and baboons, detailed genetic analyses revealed a modular character of several genes and genomic regions. While TPE genomes appear to be the most conserved genomes of uncultivable pathogenic treponemes, diversification of TPE genomes appears to be facilitated by intra-strain genome recombination events. In addition to genes with an identified modular structure, we identified additional genes that have direct or inverted repeats and thus have the potential for genetic reshuffling.

## Introduction

The infectious agent of yaws, *Treponema pallidum* subsp. *pertenue* (TPE), causes chronic infections in children and young adults, which is characterized by skin lesions including nodules and ulcerations of the skin, which is later accompanied by joint, soft tissue, and bone manifestations (reviewed in [1]). Unlike the syphilis treponemes, *Treponema pallidum* subsp. *pallidum* (TPA), TPE and *Treponema pallidum* subsp. *endemicum* (TEN, the causative agent of endemic syphilis) are transmitted between individuals mostly through direct skin contact. However, possible sexual transmission has been reported for TEN, a treponeme highly related to TPE [2–4].

Only a limited number of TPE strains/isolates have been characterized to date, mainly as a result of the uncultivable character of TPE, low number of available laboratory strains, and a limited number of clinical isolates with sufficient numbers of treponemal DNA copies per sample. However, the recent study by Edmonson et al. [5] showed a successful long-time *in vitro* cultivation of syphilis treponemes that could be also potentially applied to TPE strains. This could result in an increase in the number of characterized TPE strains. So far, seven TPE strains have been completely sequenced, including five strains of human origin (CDC-2, CDC 2575, Gauthier, Ghana-051, and Samoa D) [6,7] and two TPE strains (Fribourg-Blanc and LMNP-1) isolated from a Guinea baboon (*Papio papio*) in West Africa [8] and an olive baboon (*Papio anubis*) from Tanzania [9], respectively. In addition to these complete genomes, genomes of 6 other TPE isolates of human origin [10] and 7 from nonhuman primates have been sequenced to draft genome quality [9]. TPE strains have been shown to be highly similar to syphilis-causing strains of *T*. *pallidum* subsp. *pallidum* (TPA) [6] and to the TEN strain Bosnia A [11].

While there is an increasing understanding of genome structure and plasticity in TPA and TEN [12,13], the genome characteristics of TPE remain largely unexplored. As a result, little is known about intra-strain recombinations that occur in TPE strains and their role in genome evolution and diversification.

In this communication, we compared the complete genome sequences of two strains of TPE isolated in Indonesia to other available TPE whole genome sequences and identified regions resulting from intra-strain genome recombinations. While TPE genomes appear to be relatively conserved compared to the genomes of other uncultivable pathogenic treponemes, including TPA and TEN strains, genetic diversification of TPE genomes appears to be facilitated by intra-strain genome rearrangements.

## Material and methods

### Ethics statement

TPE strains Kampung Dalan K363 and Sei Geringging K403 originated from the study of Noordhoek *et al*. [14], where involved persons or parents of involved children gave informed consent for sample collection. No vertebrate animals were used in the study.

### Strains used in this study

Two TPE strains, Kampung Dalan K363 and Sei Geringging K403, were used in this study. TPE Kampung Dalan K363 was isolated on January 5, 1990 and TPE Sei Geringging K403 on May 14, 1990 in villages in the Pariaman region of Sumatra, Indonesia. Both TPE strains were isolated from patients having skin lesions. Skin biopsies were first homogenized in PBS and intra-dermally inoculated into the shaved inguinal areas of Syrian Golden hamsters, which were later transported to the Netherlands [14]. Hamsters that developed skin lesions were sacrificed and the inguinal lymph nodes were homogenized in PBS and inoculated into the testes of New Zealand White rabbits. Several serial passages in rabbits were performed before samples were taken for isolation of treponemal DNA. TPE strains Kampung Dalan K363 and Sei Geringging K403 were provided as DNA samples by Dr. S. Bruisten (Public Health Laboratory, Department of Infectious Diseases GGD Amsterdam) who derived the DNA samples from crude treponemal lysates which were kindly donated by Dr. G. Noordhoek who collected these samples in Indonesia and processed them in the Netherlands [14]. The determination of the number of treponemal DNA copies per μl of samples was not performed.

### Amplification of TPE genomic DNA

Total DNA of both samples was first amplified using multiple displacement amplification (REPLI-g kit, QIAGEN, Valencia, CA, USA) according to the manufacturer’s instructions. The amplified DNA was then diluted 50-times and used as a template for TPE whole genome amplification, which was performed with treponemal specific primers as described previously [6–8,11,15]. For amplification of individual amplicons (n = 278, S1 Table), PrimeSTAR GXL DNA Polymerase (Takara Bio Inc., Otsu, Japan) was used. PCR products were generated using touchdown PCR under the following cycling conditions: initial denaturation at 94°C for 1 min; 8 cycles: 98°C for 10 s, 68°C for 15 s (annealing temperature gradually reduced by 1°C/every cycle), and 68°C for 6 min; 35 cycles: 98°C for 10 s, 61°C for 15 s, and 68°C for 6 min (43 cycles in total); followed by final extension at 68°C for 7 min. All overlapping PCR products were purified using a QIAquick PCR Purification Kit (QIAGEN, Valencia, CA, USA) and mixed in equimolar amounts. Each pool of PCR products (n = 4 for each of TPE strains, S1 Table) was then used for whole genome sequencing.

### Whole genome sequencing and *de novo* assembly of the TPE genomes

Individual pools of both TPE samples were used for Illumina Nextera XT library preparation and subsequently sequenced on a MiSeq platform (2x300 bp) at the sequencing facility of CEITEC (Brno, Czech Republic). Resultant sequencing data were quality pre-processed using Trimmomatic (v0.32) [16] with a sliding window length of 4 bp and a Phred quality threshold value equal to 17. After pre-processing, sequencing reads shorter than 50 bp were removed. Sequencing results for individual pools are summarized in S2 Table.

The Illumina sequencing reads corresponding to individual pools of both TPE samples were handled separately and *de novo* assembled using SeqMan NGen v4.1.0 software (DNASTAR, Madison, WI, USA) using default parameters. A total of 222, 249, 158, and 265 contigs from the TPE Kampung Dalan K363 strain and 124, 92, 93, and 87 contigs from the TPE Sei Geringging K403 strain were obtained for Pools 1–4, respectively (S2 Table). The resulting contigs obtained from both strains were then separately aligned to the TPE Samoa D genome (CP002374.1 [6]) using Lasergene software (DNASTAR, Madison, WI, USA). In parallel, the Illumina sequencing reads corresponding to individual pools were mapped to the corresponding pool sequences (S2 Table) of the TPE Samoa D genome (CP002374.1 [6]) and both the *de novo* and reference-guided approaches were compared. All genome gaps and discrepancies were resolved using Sanger sequencing. Altogether, 11 and 6 genomic regions of the TPE Kampung Dalan K363 and TPE Sei Geringging K403 strains were Sanger sequenced, respectively. The consensual sequences for individual pools were then used to compile the complete genome sequences of both TPE strains.

To determine the number of repetitions within the *arp* (TP0433) gene, the repetitive sequences (between coordinates 462430–463157 in the TPE Samoa D genome [6]) were amplified and Sanger sequenced using the primers 32BrepF1 (5′-CGTTTGGTTTCCCCTTTGTC-3′) and 32BrepR1 (5′-GTGGGATGGCTGCTTCGTATG-3′) as described elsewhere [17]. Similarly, the repetitive sequences within the TP0470 gene (Samoa D coordinates 498895–499200) were amplified and sequenced using the primers TPI34F4 (5′-GTCTTGTGCACATTATTCAAG-3′) and TPI34R5 (5′-CTTCGTGCAACATCGCTACG-3′). The intra-strain variability, relative to the length of several G/C-homopolymeric tracts, was identified in both genomes and the prevailing length of G/C regions was used in the final genome sequences.

### Gene identification, annotation, and classification

Genes were annotated using Geneious software (v5.6.5) [18] as described previously [11] and were tagged with TPEKDK363_ and TPESGK403_ prefixes. Locus tag numbering corresponded to tag numbering for the orthologous genes annotated in the TPE Samoa D genome (CP002374.1 [6]). The TPE Samoa D genome contains one (TPESAMD_0005a) additionally annotated gene compared to the TPE Kampung Dalan K363 and TPE Sei Geringging K403 genomes. This gene is fused to TP0006 and is designated as TPEKDK363_0006 and TPESGK403_0006, respectively. Four genes (TPEKDK363_0146, TPEKDK363_0520, TPEKDK363_0812, and TPEKDK363_0856a) and eight genes (TPESGK403_0126, TPESGK403_0146, TPESGK403_0312a, TPESGK403_0435a, TPESGK403_0520, TPESGK403_0812, TPESGK403_0865, and TPESGK403_0924a) were annotated as pseudogenes in the TPE Kampung Dalan K363 and TPE Sei Geringging K403 genomes, respectively. Since the *tprK* gene showed intra-strain variability, the corresponding nucleotide positions were denoted with an “N” in the complete genome sequences. For proteins with unpredicted functions, a 150 bp-gene size limit was applied.

### Identification of genetic heterogeneity in the sequenced genomes

The identification of genetic heterogeneity was carried out as described by Strouhal et al. [7]. Briefly, individual Illumina reads were mapped to the final version of the genome sequence using SeqMan NGen (v4.1.0) software with default parameters and requiring at least a 93% read identity relative to the reference genome. For the determination of the frequency of each nucleotide in every single genome position, the haploid Bayesian method was used for SNP calculation using the same software. Individual reads supporting a less frequent allele located at the 3’-terminus (i.e., five or less nucleotides) were omitted. At least thirty independent reads from both directions were required. Nucleotide positions located within homopolymeric tracts (defined as a stretch of six or more identical nucleotides) were excluded from analysis. Chromosomal loci showing genetic heterogeneity within TPE genomes were defined as those containing more than 8% alternative reads in regions having a coverage depth greater than 100x. Candidate sites were then visually inspected using SeqMan NGen (v4.1.0) software; the *tprK* (TP0897) gene, showing intra-strain variability, was excluded from the analysis.

### Analysis of whole genome sequences

Phylogenetic trees of the TPE strains were constructed from available whole genome sequences (S3 Table) including the Samoa D (CP002374.1 [6]), CDC-2 (CP002375.1 [6]), Gauthier (CP002376.1 [6]), Fribourg-Blanc (CP003902.1 [8]), Ghana-051 (CP020365.1 [7]), CDC 2575 (CP020366.1 [7]), and LMNP-1 (CP021113.1 [9]) strains. Moreover, 6 additional draft genomes of TPE strains isolated on Solomon Islands [10] were used to determine the phylogenetic relatedness of TPE Kampung Dalan K363 and Sei Geringging K403 strains. The genome of TEN Bosnia A strain (CP007548.1 [11]) was used as an outgroup.

Whole genome alignment was constructed using SeqMan software (DNASTAR, Madison, WI, USA) and phylogenetic trees were constructed using the Maximum Likelihood method based on Tamura-Nei model [19] and with MEGA software [20]. Since there were chromosomal regions that included: (1) the *tprD* and *tprK* genes, (2) intergenic regions within both *rrn* operons, and (3) sequences in the *arp* and in the TP0470 genes, which are recombinant or repetitive in TPE strains (S3 Table), these regions were excluded from the phylogenetic analyses.

For analysis of the modular structure of the TP0136, TP0856, and TP0858 genes, additional available treponemal whole genome sequences were used including: TPA strains Nichols (CP004010.2 [21]), SS14 (CP004011.1 [21]), DAL-1 (CP003115.1 [22], Mexico A (CP003064.1 [23]), Chicago (CP001752.1 [24]), and Sea81-4 (CP003679.1 [25]) and *T*. *paraluisleporidarum* ecovar Cuniculus strain Cuniculi A (CP002103.1 [26]).

### Identification of sequentially unique *k*-mers

For each TPE genome sequence (n = 9; S3 Table), the number of canonical *k*-mers of length 9–33 nt were determined using Jellyfish software (v2.0.0) [27]. The number of unique *k*-mers saturated at a length of 17 nts and the 17-mers and longer *k*-mers were used for further evaluation. In order to determine their exact locations and their exact numbers, the detected *k*-mers were mapped to the TPE genomes using EMBOSS fuzznuc (v6.6.0) [28]. Subsequently, *k*-mers were divided into two groups with the first group comprised of *k*-mers with exactly the same numbers in all tested TPE genomes and the second group comprised of *k*-mers with different numbers in at least one TPE genome. Localization of *k*-mers in the annotated genes of each TPE genome was carried out using BEDTools intersect (v2.26.0) [29]. For each gene, the number and type of overlapping *k*-mers was determined using R (v3.4.1, packages rio v0.5.5, dplyr v0.7.3) [30]. Similarly, for each *k*-mer, the number and type of overlapping genes was determined and *k*-mers with more than a single localization in at least one genome were extracted.

### Nucleotide sequence accession numbers

The complete genome sequences from TPE Kampung Dalan K363 and TPE Sei Geringging K403 were deposited in the GenBank under accession number CP024088.1 and CP024089.1, respectively.

## Results

### Whole genome sequencing of the TPE Kampung Dalan K363 and TPE Sei Geringging K403 strains and *de novo* assembly of the genomes

Both TPE strains were sequenced using the pooled segment genomic sequencing (PSGS) protocol as previously described [6–8,11,15]. Illumina sequencing resulted in 7,545,122 paired reads and 1,241,564,236 total bases, with an average coverage depth of 1,021x for the TPE Kampung Dalan K363 genome, and 3,784,916 paired reads and 784,165,636 total bases, with an average coverage depth of 644x for the TPE Sei Geringging K403 genome. A total of 222, 249, 158, and 265 contigs for each of the pools 1–4 of the TPE Kampung Dalan K363 strain and 124, 92, 93, and 87 contigs for the 4 pools of the TPE Sei Geringging K403 strain were obtained by *de novo* assembly. Detailed characteristics of Illumina sequencing and *de novo* assembly are shown in S2 Table. The genome structures of both TPE strains were similar to other previously characterized TPE strains with no major chromosomal rearrangements. The summarized genomic features of TPE Kampung Dalan K363 and TPE Sei Geringging K403 were compared to the most closely related TPE Samoa D genome (CP002374.1 [6]). Details are shown in Table 1.

**Table 1:** Basic characteristics of the TPE Kampung Dalan K363 and TPE Sei Geringging K403 genomes and their comparison to the published TPE Samoa D genome.

Genome parameter	TPE Kampung Dalan K363	TPE Sei Geringging K403	TPE Samoa D^a^
GenBank Accession No.	CP024088.1	CP024089.1	CP002374.1
Genome size	1,139,764 bp	1,139,464 bp	1,139,330 bp
G+C content	52.78%	52.77%	52.80%
No. of predicted genes	1124 including 54 untranslated genes^b^	1124 including 54 untranslated genes^b^	1125 including 54 untranslated genes^b^
Sum of the intergenic region length (% of the genome length)	53,026 bp (4.65%)	53,213 bp (4.67%)	52,844 bp (4.64%)
Average/median gene length	981.4/834.0 bp	981.5/834.0 bp	980.3/831.0 bp
Average/median gene length of genes with unknown function	819.1/636.0 bp	821.1/640.5 bp	843.4/657.0 bp
No. of genes encoded on plus/minus DNA strand	599/525	599/525	600/525
No. of annotated pseudogenes	4	8	6
No. of tRNA loci	45	45	45
No. of rRNA loci	6 (2 operons)	6 (2 operons)	6 (2 operons)
No. of ncRNAs	3	3	3

^a^ [6]

^b^ The TPE Samoa D genome contains one (TPESAMD_0005a) additionally annotated gene compared to the TPE Kampung Dalan K363 and TPE Sei Geringging K403 genomes where this gene is fused to TP0006 and is designated as TPEKDK363_0006 and TPESGK403_0006, respectively.

### Analysis of whole genome sequences

The genomes of TPE Kampung Dalan K363 and TPE Sei Geringging K403 strains differed in the number of repetitions within the *arp* (TP0433) and TP0470 genes (S3 Table). The TPE Kampung Dalan K363 strain contained 4 and 35 repetitions in the *arp* and TP0470 genes, respectively, while the TPE Sei Geringging K403 strain contained 2 and 28 repetitions within these genes, respectively. In both strains, the same repeat motif (Type II) within the *arp* gene was identified as previously shown in other TPE strains [31]. Both TPE genomes showed the same constitution of intergenic spacer regions within the *rrn* operons, i.e., tRNA-Ile/tRNA-Ala pattern [13,32] (S3 Table), and both contained the *tprD2* allele in the *tprD* locus [33] (S3 Table). Moreover, both TPE genomes differed in the sequences of the *tprK* gene variable regions [34–37].

The whole genome sequences of the TPE Kampung Dalan K363 and TPE Sei Geringging K403 strains were analyzed with respect to the occurrence of nucleotide diversity between both strains. As a result, the genomes differed in 38 single nucleotide positions (S4 Table). In addition, both genomes differed in 18 nucleotide positions within the TP0858 gene. Moreover, there were differences in both genomes in the number of 2 nt-long (TG) and 9 nt-long (TCCTCCCCC) repetitive sequences between coordinates 390964–390969 and 1051995–1052003 (according to the TPE Samoa D genome [6]), respectively. The genome of the TPE Kampung Dalan K363 strain contained 2 and 2 of these repetitive sequences, while the TPE Sei Geringging K403 genome contained 3 and 1 of these repetitions (S4 Table), respectively. Since both TPE strains Kampung Dalan K363 and Sei Geringging K403 underwent serial passages in hamsters and rabbits after their isolation from human patients, the identified genetic differences resulted either from the cultivation experiments in animals or were already present during infection of humans.

**Fig 1:**
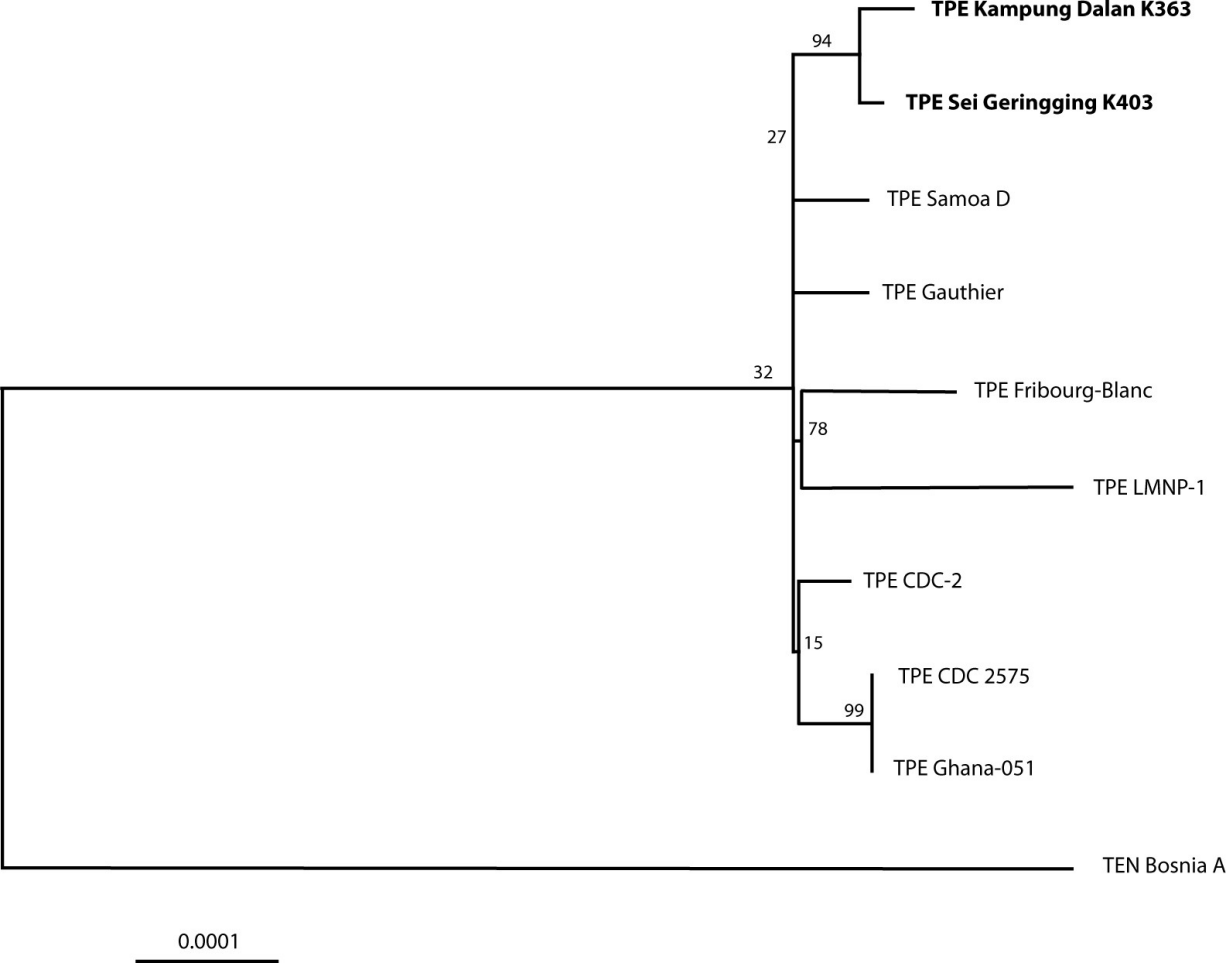
A phylogenetic tree constructed from the whole genome sequence alignment of available TPE and TEN complete genome sequences (S3 Table). The tree was constructed using the Maximum Likelihood method based on the Tamura-Nei model [19] and MEGA software [20]. The bar scale corresponds to a difference of 0.0001 nucleotides per site. Bootstrap values based on 1,000 replications are shown next to the branches. There were a total of 1,189 informative positions in the final dataset. Genome sequence of TEN strain Bosnia A was used as an outgroup. Both TPE strains of Indonesian origin were highly related to each other when compared to the genetic diversity detected among other, previously characterized TPE strains. Both intergenic spacer regions within the *rrn* operons, the *tprK* (TP0897) and *tprD* (TP0131) gene sequences and the repetitive sequences within the *arp* (TP0433) and TP0470 genes were omitted from the analysis. The TPE Kampung Dalan K363 and TPE Sei Geringging K403 strains are shown in bold.

As revealed by our phylogenetic analyses, the TPE Kampung Dalan K363 and TPE Sei Geringging K403 strains clustered together and were more distantly related to other complete genomes of TPE strains of human or baboon origin (Fig 1). In contrast to the Indonesian strains used in this study, all other TPE strains originated from Africa with the exception of the TPE Samoa D strain, which was isolated in Western Samoa in 1953 (S3 Table). Nevertheless, additional phylogenetic analysis including the recently published TPE draft genome sequences from 6 individuals from Solomon Islands [10] did not show clustering of TPE Kampung Dalan K363 and TPE Sei Geringging K403 with these strains (S1 File). While all Solomon Island isolates [10] clustered together and were more closely related to TPE Samoa D, TPE Kampung Dalan K363 and TPE Sei Geringging K403 belonged to distinct cluster.

### Intra-strain heterogeneity in the TPE Kampung Dalan K363 and TPE Sei Geringging K403 genomes

The TPE Kampung Dalan K363 and TPE Sei Geringging K403 genomes were inspected for the presence of genetic intra-strain heterogeneity [38]. While the genome of the TPE Kampung Dalan K363 strain contained 3 intra-strain heterogeneous sites, the genome of the TPE Sei Geringging K403 strain harbored only a single such site (S5 Table). The TPE Kampung Dalan K363 strain contained heterogeneous sites in genes TP0448 (encoding uracil phosphoribosyltransferase), TP0488 (coding for methyl-accepting chemotaxis protein), and TP1032 (encoding hypothetical protein). In the TPE Sei Geringging K403 strain, a single heterogeneous site was found in the TP0363 gene, encoding chemotaxis protein CheA, which is a sensor histidine kinase. All four heterogeneous sites resulted in amino acid replacements in the corresponding proteins (S5 Table).

In both TPE genomes, intra-strain variability in the length of G/C-homopolymeric tracts was identified as previously shown in other treponemal genomes [7,39,40]. Based on the prevailing length of G/C regions in the final genome sequences, 16 out of 44 such regions were found to be different when comparing the TPE Kampung Dalan K363 and TPE Sei Geringging K403 genomes (S6 Table). Therefore, four genes (TPESGK403_0126, TPESGK403_0312a, TPESGK403_0865, and TPESGK403_0924a) were annotated as pseudogenes in the TPE Sei Geringging K403 genome (S6 Table).

### Modular structure of the TP0856 and TP0858 genes

**Fig 2:**
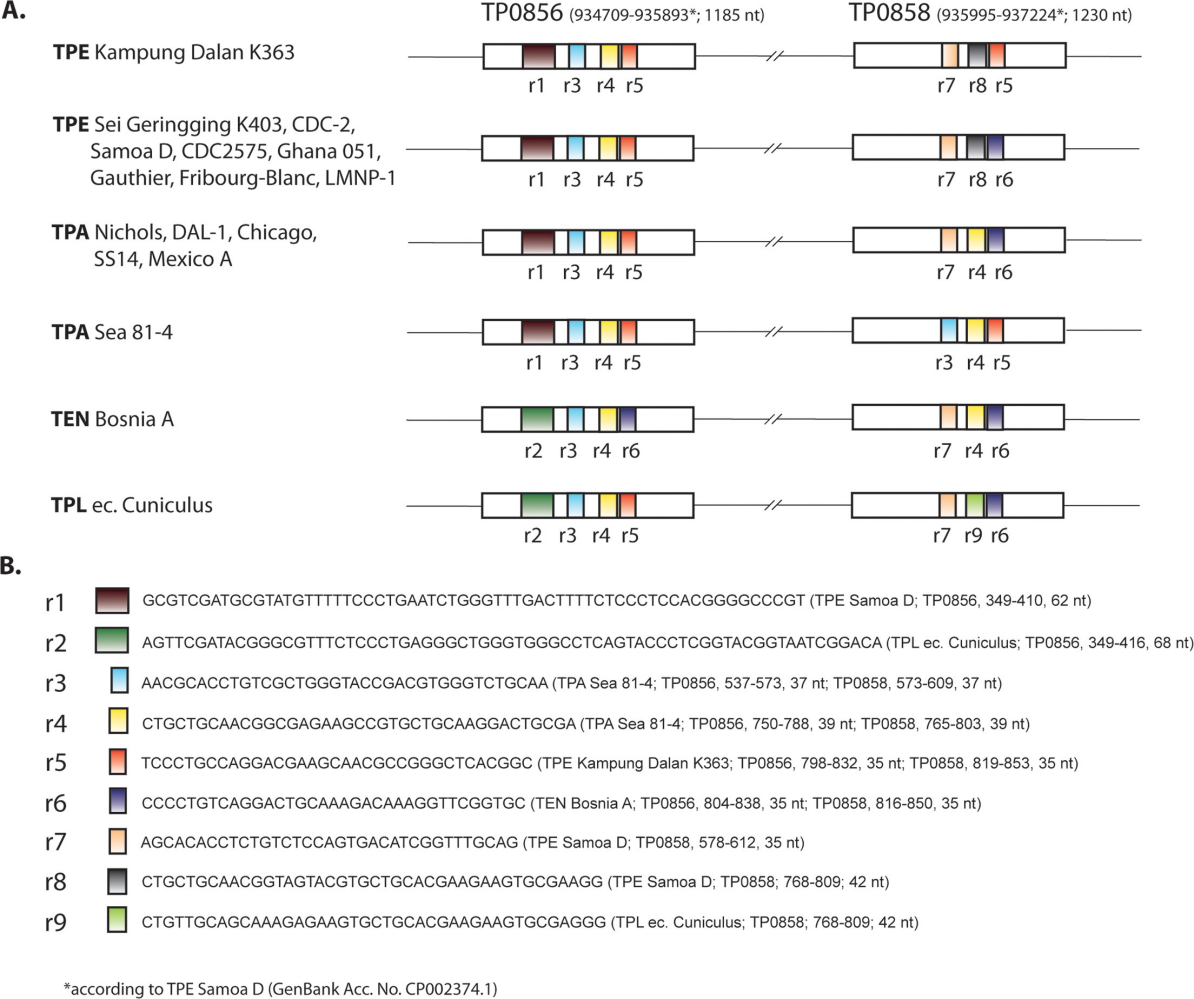
The modular structure of the TP0856 and TP0858 genes among completely sequenced treponemal strains. **A.** Please note the differences between sequence patterns of TPE Kampung Dalan K363 and the other TPE strains, the TPA Sea81-4 and the other TPA strains, and TPE and TPA/TEN strains in TP0858 gene. The r3 sequence (in TPA Sea81-4), r4 sequence (in TPA and TEN strains), r5 sequence (in Kampung Dalan K363 and TPA Sea81-4) and r6 sequence (in TEN Bosnia A) within TP0858 or TP0856 genes resulted probably from intra-strain recombination events. **B.** A list of repetitive (r3, r4, r5, r6) and non-repetitive (unique) sequences (r1, r2, r7, r8, r9).

Although both Indonesian TPE strains were highly related to each other, a relatively long stretch of nucleotide differences in the TP0858 gene sequence of both analyzed strains suggests a potential recombination event. Compared to the TP0858 sequence of the TPE Sei Geringging K403 strain (which was similar to the other TPE strains), the TP0858 sequence in the TPE Kampung Dalan K363 strain differed in 18 nucleotide positions (coordinates 819–853 in the TP0858 gene of the TPE Samoa D [6]). Moreover, the nucleotide sequence present in the TP0858 gene of the TPE Kampung Dalan K363 strain (i.e., r5 sequence; see Fig 2) was found to be identical with the one found between coordinates 798–832 in the TP0856 gene (TPE Samoa D gene coordinates). Interestingly, the same sequence (i.e., r5 sequence; see Fig 2) was detected also in the TP0858 gene of the TPA Sea 81–4 (coordinates 819–853 according to the TPE Samoa D TP0858 gene). Analysis of additional treponemal genomes revealed that the TEN Bosnia A strain contained identical sequences in the above described regions of both the TP0856 and TP0858 genes (i.e., r6 sequence; see Fig 2), even though these sequences in the TEN Bosnia A strain and the TPE Kampung Dalan K363 strain differed (Fig 2). Upstream of this sequence, between coordinates 768–809 in the TP0858 gene (TPE Samoa D gene coordinates), there was a 42 nt-long DNA region (i.e., r8 and r4 sequences; see Fig 2) showing identical sequences within TPE and TPA/TEN strains, respectively, but different in the TPE and TPA/TEN comparison (Fig 2). In addition, the TPA Sea81-4 strain contained identical sequences (i.e., r3 sequence; see Fig 2) in both the TP0856 and TP0858 genes between coordinates 538–573 and 579–612 (TPE Samoa D gene coordinates), respectively. The modular structure of the TP0856 and TP0858 genes comprising all completely sequenced treponemal strains is depicted in more detail in Fig 2.

Protein sequence analyses revealed that the repetitive modules found within both TP0856 and TP0858 genes (e.g., r3 in TPA Sea81-4, r4 in TPA and TEN strains, r5 in TPE Kampung Dalan K363 and TPA Sea81-4, and r6 in TEN Bosnia A) used the same reading frame and therefore yielded the same amino acid sequence in both TP0856 and TP0858 proteins. Protein function analysis of TP0856 and TP0858 revealed presence of UPF0164, an uncharacterized protein family found only among *T*. *pallidum* strains. Members of this protein family belong to the membrane beta barrel superfamily. No motifs were found within these genes using the Motif search (https://www.genome.jp/tools/motif) and Pfam, NCBI-CDD and PROSITE Profile databases. However, as described recently [41], TP0856 and TP0858 proteins showed structural similarity to FadL, a long fatty acid transporter.

In addition, the sequences of TP0856 and TP0858 were analyzed by I-TASSER server [42] to predict the protein structure. The analyses revealed that most of the variable sites (i.e., r1, r2, r4, r5, r6, r8 and r9) of TP0856 and TP0858 represent coil sequences at the outer surface of β-barrels suggesting that these protein loci are exposed to the external milieu. The detailed overview of predicted structure for module sequences in TP0856 and TP0858 genes are shown in S7 Table.

### Modular structure of the TP0136 gene

**Fig 3:**
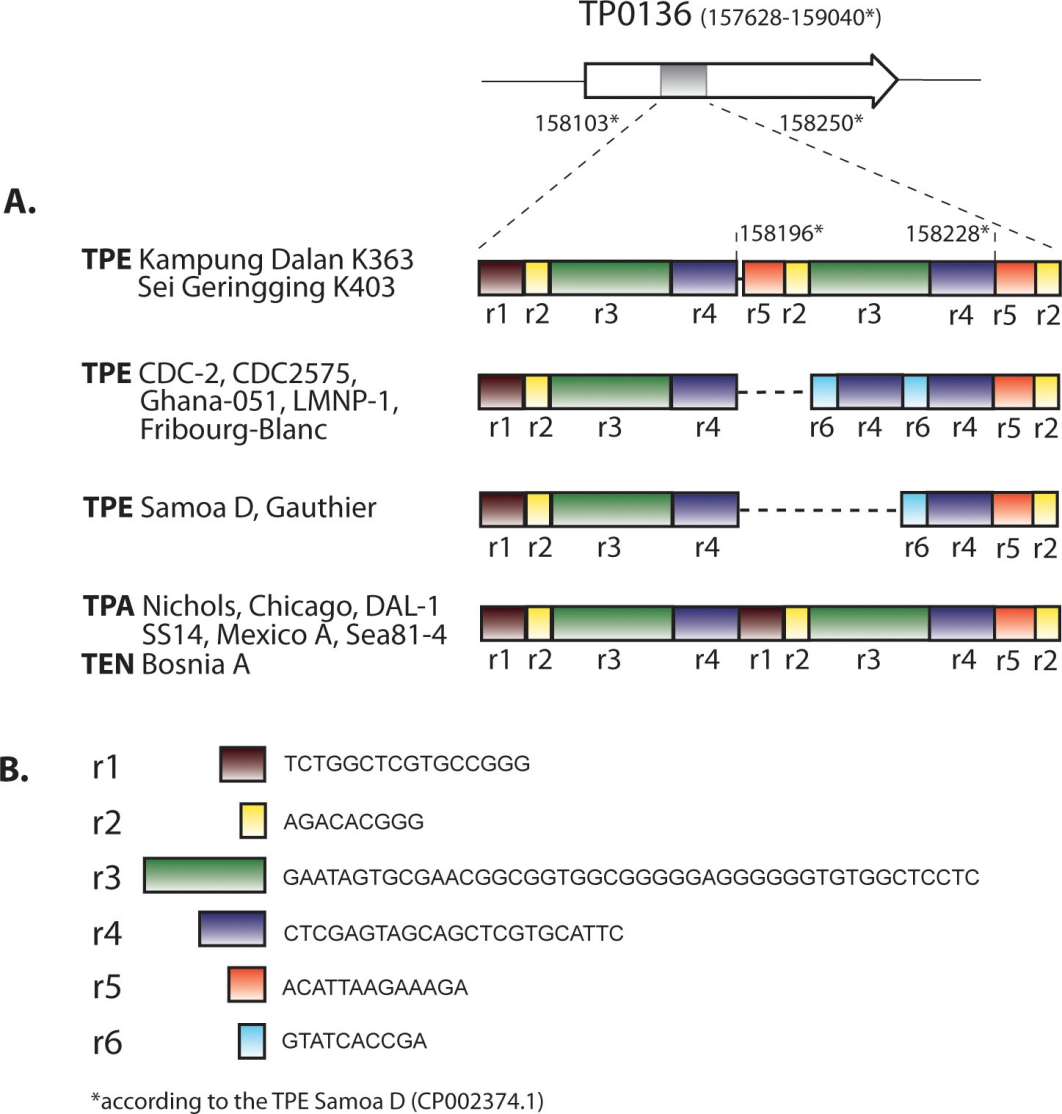
**A.** Modular structure of the TP0136 gene of the TPE Kampung Dalan K363, TPE Sei Geringging K403 and additional TPE, TEN, and TPA strains. While the TPE Kampung Dalan K363 and TPE Sei Geringging K403 strains resemble TPA strains, other TPE strains showed a deleted version of the TP0136 gene (Samoa D and Gauthier) or a deleted version with a duplicated subregion r6 and r4 (CDC-2, CDC 2575, Ghana-051, Fribourg-Blanc, and LMNP-1). The sequence of the TP0136 gene from the *Treponema paraluisleporidarum* ecovar Cuniculus strain Cuniculi A was not included in this analysis since this gene locus contains a genetically different sequence, which is identical (99.7% at the DNA level) to the TP0133 gene sequence. **B.** A list of repetitive sequences (r1, r2, r3, r4, r5, r6).

An alignment of both whole genome sequences of the TPE Kampung Dalan K363 and TPE Sei Geringging K403 strains revealed striking sequence differences in the TP0136 gene compared to other TPE strains. A detailed analysis of treponemal TP0136 gene orthologs identified a modular structure in the region between coordinates 158103–158250 (coordinates according to the TPE Samoa D genome [6]; see Fig 3). While in the TPE Samoa D and TPE Gauthier strains, the DNA region between coordinates 158196–158228 is represented by a 33-nt long sequence (i.e., r6 and r4 sequences; see Fig 3), in TPE strains CDC-2, CDC 2575, Fribourg-Blanc, and LMNP-1, the same region contains an additional copy of this 33-nt long sequence (Fig 3). In contrast, the TPE Kampung Dalan K363 and TPE Sei Geringging K403 strains contain within this region a duplicated segment from coordinates 158229–158250 (i.e., r5 and r2 sequences; see Fig 3), followed by another duplicated sequence from positions 158128–158195 (i.e., r3 and r4 sequences; see Fig 3). The sequence of the TP0136 gene from the TPE Kampung Dalan K363 and TPE Sei Geringging K403 strains thus resembles the TP0136 gene sequences found in TPA strains (Fig 3), where two 96 nt-long repetitions are present (i.e., comprising r1-r2-r3-r4 sequences). The modular structure of the TP0136 gene comprising all completely sequenced treponemal strains is depicted in more detail in Fig 3.

### Prediction of treponemal genes with a modular structure in TPE genomes

**Fig 4:**
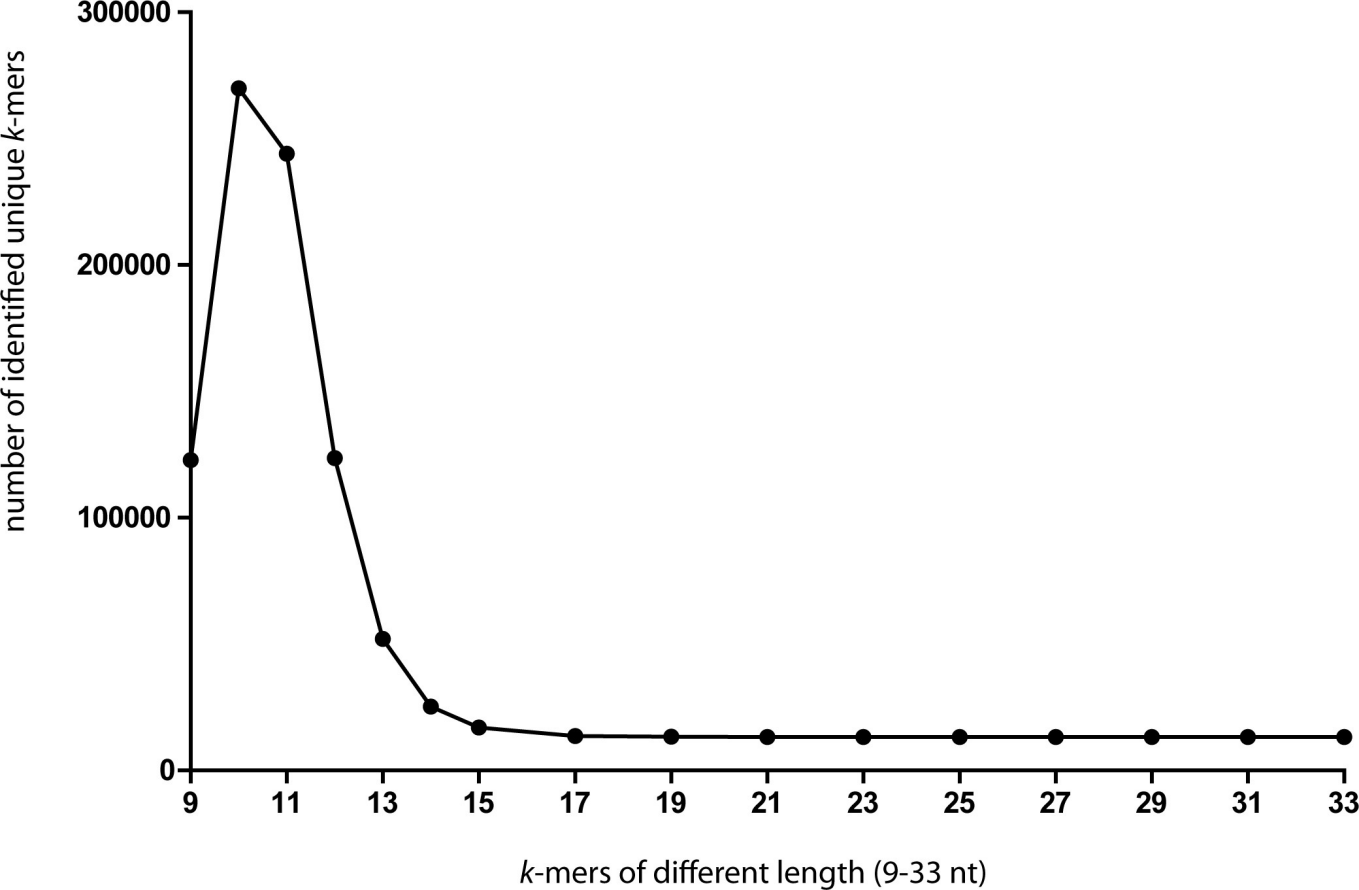
Number of identified *k*-mers of different length (9–33 nt) derived from TPE genome sequences. The number of unique *k*-mers saturated at a length of 17 nts, which was subsequently used as the cutoff length for further evaluations.

**Fig 5:**
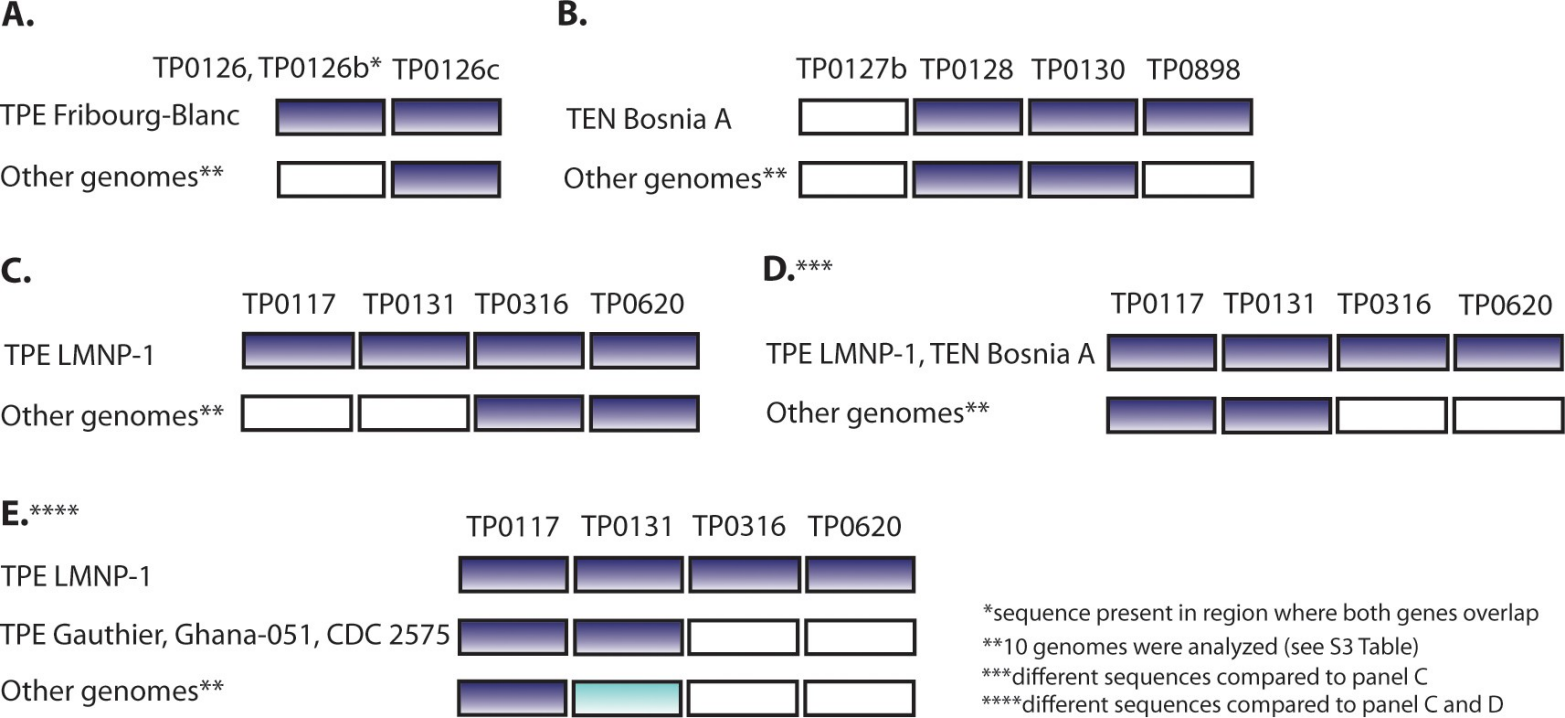
A schematic representation of examples of detected modular structure in several treponemal genes. **A.** The modular structure of TP0126/TP0126b and TP0126c genes (coordinates 148661–148691 and 149217–149247, respectively, according to the TPE Samoa D genome [6]). The TPE Fribourg-Blanc strain has identical sequences between regions where the TP0126 and TP0126b genes overlap and in TP0126c, while other analyzed genomes have different sequences in the TP0126/TP0126b. **B.** The modular structure of the TP0127b, TP0128, TP0130, and TP0898 genes (coordinates 150108–150120, 150605–150619, 151643–151659, and 977776–977789, respectively, according to the TPE Samoa D genome [6]). The TEN Bosnia A strain has identical sequences in regions of TP0128, TP0130, and TP0898 genes, while other genomes have the same sequences between regions of the TP0128 and TP0130 genes and between the TP0127b and TP0898 genes. **C.** The modular structure of the TP0117, TP0131, TP0316, and TP0620 genes (coordinates 134610–134655, 152056–152101, 331882–331927, and 672661–672706, respectively, according to the TPE Samoa D genome [6]). The TPE LMNP-1 strain has identical sequences in all of these genes while other genomes have the same sequences between regions of the TP0117 and TP0131 genes and between the TP0316 and TP0620 genes. **D.** The modular structure of the TP0117, TP0131, TP0316, and TP0620 genes (coordinates 134676–134672, 152122–152138, 331948–331964, and 672727–672743, respectively, according to the TPE Samoa D genome [6]). The TPE LMNP-1 and TEN Bosnia A strains have identical sequences in all of these genes while other genomes have the same sequences between regions of the TP0117 and TP0131 genes and between the TP0316 and TP0620 genes. **E.** The modular structure of the TP0117, TP0131, TP0316, and TP0620 genes (coordinates 134780–134804, 152226–152250, 332052–332076, and 672831–672855, respectively, according to the TPE Samoa D genome [6]). The TPE LMNP-1 strain has identical sequences in all these genes while TPE strains Gauthier, CDC 2575, and Ghana-051 have identical sequences between regions of the TP0117 and TP0131 genes and between the TP0316 and TP0620 genes. The remaining genomes have the same sequences between the TP0316 and TP0620 genes.

To systematically detect genes showing a modular genetic structure, a whole genome analysis of the presence of direct or inverted repeats of 17 or more nucleotides in length was performed. The length of 17 or more nucleotides was based on an analysis of identified sequentially unique *k*-mers present in TPE genomes. Starting with *k*-mers 9 nts in length, the number of different *k*-mers increases with the length of *k*-mers until it reaches a maximum at 11 nts and then decreasing (Fig 4). In *k*-mers 17 nt in length, the number of detected different *k*-mers remains stable and therefore this length of *k*-mers was selected for identification of positions and multiplicity of these *k*-mers in the TPE strain genomes. The results of position- and multiplicity-mapping of *k*-mers are summarized in Table 2. Besides *tpr* genes (*tprCDEFGIJK*), a frequent presence of repeats was found in the region spanning the TP0126–TP0136 genes, and in TP0856, TP0858, and TP0896 genes. Examples of other treponemal genes showing a different modular structure are presented in Fig 5.

**Table 2:** The list of detected genes with direct or inverted repeats of 17 or more nucleotides in length.

Gene	Gene name	Identical repeat found in	Prediction of protein function
TP0117	*tprC*	*tprDFI*	Tpr protein C
TP0126abc		TP0129, TP0129ab	hypothetical protein
TP0129		TP0126abc, TP0129ab	glutamate 5-kinase
TP0130		*tprK*	repeat protein K^a^
TP0131	*tprD*	*tprCFI*	Tpr protein D
TP0136		TP0133, TP0134	fibronectin binding protein, outer membrane protein^a^
TP0136a		*tprK*	hypothetical protein
TP0313	*tprE*	*tprGJ*	Tpr protein E
TP0316	*tprF*	*tprCDI*	Tpr protein F
TP0317	*tprG*	*tprEJ*	Tpr protein G
TP0620	*tprI*	*tprCDF*	Tpr protein I
TP0621	*tprJ*	*tprGE*	Tpr protein J
TP0856		TP0858	lipoprotein^a^
TP0896		TP0126a, *tprK*	ATP synthase CF1 alpha subunit^a^

^a^protein predictions by Naqvi et al. [43]

## Discussion

Two TPE strains isolated in 1990 from villages in the Pariaman region of Sumatra, Indonesia, were completely sequenced in this study using the pooled segment genomic sequencing (PSGS) approach. This approach allowed assembly and compilation of complete genome sequences without gaps or ambiguous nucleotide positions. The only exception was the variable regions within the *tprK* gene where consensus sequences were not determined due to intra-strain nucleotide sequence variability [34–37]. Both Indonesian TPE strains, i.e., Kampung Dalan K363 and Sei Geringging K403, were genetically related to each other and both strains were more distantly related to other, previously characterized TPE strains.

Most of the available complete genome sequences of TPE strains originated in Africa except for the TPE Kampung Dalan K363 and Sei Geringging K403 strains that were isolated in Indonesia [14] and the TPE Samoa D strain that was isolated from the Samoan Islands in the central South Pacific, forming part of Polynesia [44]. This opens the question of whether TPE strains differ with respect to their geographical origin as shown by molecular typing studies of TPA [13]. A recent paper on TPE isolates sequenced from the Solomon Islands revealed 8 draft genome TPE sequences from 6 patients [10] showing that the Solomon Islands genome sequences represented a discrete TPE clade that was distinct from all previously sequenced TPE strains. Nevertheless, the phylogenetic analysis including also TPE strains from Solomon Islands [10] did not show clustering of TPE Kampung Dalan K363 and TPE Sei Geringging K403 with these strains (S1 File) despite their close geographical origin. However, the draft genome status of TPE Solomon Islands strains needs to be taken into account in the interpretation of the phylogeny shown in S1 File. Interestingly, the genetic features within the TP0858 gene of the TPE Kampung Dalan K363 strain, presented in this study, was similar to those found in all of the Solomon Islands isolates. This comprised a short sequence within the TP0858 gene that was conserved in all the Solomon Islands isolates suggesting that this sequence is representative of isolates from the South Pacific region [10]. Moreover, this sequence is a part of the reverse primer binding site within the TP0858 gene, which was the target of a PCR assay designed by Chi and colleagues [45], which leads to false-negative PCR results on samples with this recombination [10,46].

A limited amount of genetic diversity within individual TPE strains was found in this study. Although it has been shown that the number of identified intra-strain heterogeneous sites correlates positively with the average depth of sequencing coverage, these genomes revealed just one and three such sites, although the average depth of sequencing coverage was well above 600x. Čejková et al. [38] proposed that the number of heterogeneous sites also reflects *T*. *pallidum* subspecies classification, where the majority of heterogeneous sites were found among TPA strains and not among TPE strains. This work appears to be consistent with this prediction same as the recently sequenced genomes of TPE strains Ghana-051 and CDC 2575, which showed a relatively limited number of heterogeneous sites (n = 13, n = 5; respectively) [7]. As shown in previous studies, all the alternative alleles identified in this study encoded non-synonymous amino acid replacements, suggesting an adaptive character for this genetic variability [38].

In general, pathogenic treponemes comprising TPA, TPE, and TEN strains or isolates, lack mobile genetic elements including pathogenicity islands, prophages, and plasmids [12,13]. It was long believed that the lack of mobile genetic elements is related to the absence of genetic recombination both within and between treponemal strains. Yet, due to recent accumulation of genetic data, treponemes appear to recombine genetic material both within and between genomes. One of the first observations describing intra-strain genetic recombinations (recombinations within genomes) came from studies on the *tprK* gene, which shows increasing variability during the course of human infection [35,36]. The underlying mechanism here is gene conversion using sequences from the flanking regions of *tprD* [34]. Later, Gray et al. [47] demonstrated that intra-genomic recombination has played a significant role in the evolution of *tpr* genes (*tprCDIGJK*), which have evolved through gene duplication and gene conversion. As an example, the occurrence of *tprD* and *tprD2*, both found within TPA clusters (Nichols-like and SS14-like) and within TPE strains [33], suggests a gene conversion mechanism in copying the *tprC* allele (that is identical with the *tprD* allele) to the *tprD* locus [33,47]. Similarly, the TP0136 locus of the *Treponema paraluisleporidarum* ecovar Cuniculus strain Cuniculi A contains an almost identical copy of the TP0133 gene sequence, suggesting the same mechanism as for *tprD*/*tprD2* allele alternation [13,26]. A similar situation was recently found in TPE samples isolated on Lihir Island, Papua New Guinea [48], where the TP0136 allele also had an intriguing sequence identity to the TP0133 gene. The authors proposed a possible interstrain recombination between treponemal species, however, intra-strain recombination by copying the TP0133 allele to the TP0136 locus appears to be more plausible. As shown in Čejková et al. [32], two rRNA (*rrn*) operons occurred in two different *rrn* spacer patterns (i.e., tRNA-Ala/tRNA-Ile and tRNA-Ile/tRNA-Ala patterns) and these variants were found independently of species/subspecies classification, time, and geographical source of the treponemal strains, suggesting the existence of reciprocal recombination in treponemes. Besides intra-genomic recombination events, traces of interstrain (intergenomic) recombination between TPA, TPE, and TEN strains have been proposed for several genetic loci [3,11,23].

Comparisons of genome sequences of the TPE Kampung Dalan K363 and TPE Sei Geringging K403 strains as well as analysis of other TPE strains revealed a modular structure for at least three gene loci including TP0136, TP0856, and TP0858, suggesting that the recombination within treponemal genomes can result in substantial changes in gene and protein sequences. Further systematic analyses revealed additional gene loci with a modular genetic structure that differ in certain strain(s) compared to others, these genes included TP0126, TP0126b, TP0126c, TP0127b, TP0128, TP0130, TP0898, and *tprCDFI* (TP0117, TP0131, TP0316, and TP0620), indicating that this mechanism, which enables genetic diversification, is quite common in treponemal genomes. Moreover, there were additional genes identified that have direct or inverted repeats (summarized in Table 2) and thus have the potential for genetic reshuffling. The analysis of these genes revealed that these loci were limited to specific and relatively short genomic regions. In addition, these regions were often found in paralogous gene families including *tpr* genes (*tprCDEFGIJK*), the paralogous family of TP0133, TP0134, TP0136, and TP0462 genes, and the paralogous family of TP0548, TP0856, TP0858, TP0859, and TP0865 genes.

As a consequence of the inherent variability of these paralogous families, restriction fragment length polymorphism (RFLP) analysis of the *tprE* (TP0313), *tprG* (TP0317), and *tprJ* (TP0621) genes became a part of the CDC-typing scheme that determines, in addition to the *tprEGJ* RFLP pattern, a number of 60-bp tandem repeats within *arp* (TP0433) [49]. Interestingly, members of two additional paralogous families including TP0136 and TP0548 are targets of sequencing-based molecular typing [50–55].

Similar to the TP0136 protein, TP0856 and TP0858 are predicted lipoproteins [43]. Structure similarity of TP0856 and TP0858 proteins to FadL, a long chain fatty acid transporter, was recently published [41] and both proteins are members of a FadL-like family (TP0548, TP0856, TP0858, TP0859, TP0865) found in *T*. *pallidum*. Moreover, most of the variable sites (i.e., r1, r2, r4, r5, r6, r8 and r9) of TP0856 and TP0858 were located in loops suggesting that these protein loci are exposed to the external milieu. The TP0136 gene has been shown to have heterogeneous sequences among *T*. *pallidum* strains [56,57]. Moreover, the TP0136 lipoprotein was demonstrated to be exposed on the surface of the bacterial outer membrane and was shown to bind to the extracellular matrix glycoproteins fibronectin and laminin [56]. Immunization with recombinant TP0136 delayed ulceration in experimentally infected rabbits but did not prevent infection or the formation of skin lesions [56]. The NH_2_-terminus of the TP0136 protein comprises a region with a modular structure overlapping the major fibronectin binding activity domain [57]. The modular structure was identified within the two 96 nt-long repetitions that are present in TPA strains and in the TEN Bosnia A strain. Interestingly, the sequence of the TP0136 gene in the TPE Kampung Dalan K363 and TPE Sei Geringging K403 strains resembled the TP0136 gene sequences found in TPA strains representing a new molecular type in the yaws MLST typing scheme [48]. Moreover, the specific insertion in TP0136 in the DAL-1 genome [22] was predicted to contain donor sequences for the *tprK* gene of *T*. *pallidum* [58].

In the case of TprC protein, one of the predicted antigenic epitopes on the 3D predicted structure, E3 (residues 575–583), partially overlaps with a recombinant region [59,60]. Our findings are therefore consistent with relatively frequent genetic recombinations operating at certain treponemal loci and these recombinations likely result in novel amino acid sequences exposed to the external milieu.

In summary, although TPE genomes appear to be the most conserved genomes of the uncultivable pathogenic treponemes [12,13], diversification of TPE genomes appears to be facilitated by intra-strain genome recombination events and rearrangements. Analysis of additional genomes will likely reveal more potential recombinations in the future.

## Supporting information

S1 Table PSGS (Pool Segment Genome Sequencing) approach—list of treponeme-specific primers used for the amplification of TP intervals of the TPE Kampung Dalan K363 and TPE Sei Geringging K403 strains. (XLSX)

S2 Table Next-generation sequencing statistics for the TPE Sei Geringging K403 and TPE Kampung Dalan K363 genomes. (XLSX)

S3 Table *Treponema* strains/genomes used in analyses. (XLSX)

S4 Table Nucleotide differences between the TPE Kampung Dalan K363 and TPE Sei Geringging K403 genomes. *tprK* (TP0897) and TP0858 genes were excluded from the analysis.
(XLSX)

S5 Table Intra-strain heterogeneity found in the TPE Kampung Dalan K363 and TPE Sei Geringging K403 genomes. Minor alleles with a frequency over 8% and coverage depth over 100x are shown. The *tprK* (TP0897) gene was excluded from the analysis.
(XLSX)

S6 Table Differences in G/C-homopolymeric tracts when comparing the TPE Kampung Dalan K363 and TPE Sei Geringging K403 genomes. (XLSX)

S7 Table The overview of predicted structure for module sequences in TP0856 and TP0858 genes. (PDF)

S1 File A tree constructed from the whole genome sequence alignment of available TPE and TEN genome sequences including also the draft genomes of TPE strains isolated on Solomon Islands [10]. The tree was constructed using the Maximum Likelihood method based on the Tamura-Nei model [19] and MEGA software [20]. The bar scale corresponds to a difference of 0.0001 nucleotides per site. Bootstrap values based on 1,000 replications are shown next to the branches. There were a total of 1,104,654 positions in the final dataset. Both TPE strains of Indonesian origin were highly related to each other when compared to the genetic diversity detected among other, previously characterized TPE strains. TPE strains from Solomon Islands [10] did not show clustering of TPE Kampung Dalan K363 and TPE Sei Geringging K403 with these strains despite their close geographical origin. Draft genome sequences from study by Marks et al. [10] are marked by prefix ERR and can be accessible from GenBank database using the corresponding name e.g. ERR1470334.
(PDF)

## References

[1] Giacani L, Lukehart SA. The endemic treponematoses. Clin Microbiol Rev. 2014;27: 89–115. 10.1128/CMR.00070-13 24396138 PMC3910905

[2] Grange PA, Mikalová L, Gaudin C, Strouhal M, Janier M, Benhaddou N, et al *Treponema pallidum* 11qj subtype may correspond to a *Treponema pallidum* subsp. *endemicum* strain. Sex Transm Dis. 2016;43: 517–518. 10.1097/OLQ.0000000000000474 27419817

[3] Mikalová L, Strouhal M, Oppelt J, Grange PA, Janier M, Benhaddou N, et al Human *Treponema pallidum* 11q/j isolate belongs to subsp. *endemicum* but contains two loci with a sequence in TP0548 and TP0488 similar to subsp. *pertenue* and subsp. *pallidum*, respectively. PLoS Negl Trop Dis. 2017;11: e0005434 10.1371/journal.pntd.0005434 28263990 PMC5354452

[4] Noda AA, Grillová L, Lienhard R, Blanco O, Rodríguez I, Šmajs D. Bejel in Cuba: molecular identification of *Treponema pallidum* subsp. *endemicum* in patients diagnosed with venereal syphilis. Clin Microbiol Infect. 2018 2 15 pii: S1198-743X(18)30154-X. 10.1016/j.cmi.2018.02.006 29454847

[5] Edmondson DG, Hu B, Norris SJ. Long-term in vitro culture of the syphilis spirochete *Treponema pallidum* subsp. *pallidum*. MBio 2018;9: e01153–18. 10.1128/mBio.01153-18 29946052 PMC6020297

[6] Čejková D, Zobaníková M, Chen L, Pospíšilová P, Strouhal M, Qin X, et al Whole genome sequences of three *Treponema pallidum* ssp. *pertenue* strains: yaws and syphilis treponemes differ in less than 0.2% of the genome sequence. PLoS Negl Trop Dis. 2012;6: e1471 10.1371/journal.pntd.0001471 22292095 PMC3265458

[7] Strouhal M, Mikalová L, Havlíčková P, Tenti P, Čejková D, Rychlík I, et al Complete genome sequence of two strains of *Treponema pallidum* subsp. *pertenue* from Ghana, Africa: identical genome sequences in samples isolated more than 7 years apart. PLoS Negl Trop Dis. 2017;11: e0005894 10.1371/journal.pntd.0005894 28886021 PMC5607219

[8] Zobaníková M, Strouhal M, Mikalová L, Čejková D, Ambrožová L, Pospíšilová P, et al Whole genome sequence of the *Treponema* Fribourg-Blanc: unspecified simian isolate is highly similar to the yaws subspecies. PLoS Negl Trop Dis. 2013;7: e2172 10.1371/journal.pntd.0002172 23638193 PMC3630124

[9] Knauf S, Gogarten J, Schuenemann VJ, De Nys HM, Duex A, Strouhal M, et al. African nonhuman primates are infected with the yaws bacterium Treponema pallidum subsp. pertenue; 2017. Preprint. Available from: bioRxiv. doi.org/10.1101/135491. 10.1038/s41426-018-0156-4 PMC6143531 30228266

[10] Marks M, Fookes M, Wagner J, Butcher R, Ghinai R, Sokana O, et al Diagnostics for yaws eradication: insights from direct next generation sequencing of cutaneous strains of *Treponema pallidum*. Clin Infect Dis. 2017 10 16 10.1093/cid/cix892 29045605 PMC5848336

[11] Štaudová B, Strouhal M, Zobaníková M, Čejková D, Fulton LL, Chen L, et al Whole genome sequence of the *Treponema pallidum* subsp. *endemicum* strain Bosnia A: the genome is related to yaws treponemes but contains few loci similar to syphilis treponemes. PLoS Negl Trop Dis. 2014;8: e3261 10.1371/journal.pntd.0003261 PMC4222731 25375929

[12] Šmajs D, Norris SJ, Weinstock GM. Genetic diversity in *Treponema pallidum*: implications for pathogenesis, evolution and molecular diagnostics of syphilis and yaws. Infect Genet Evol. 2012;12: 191–202. 10.1016/j.meegid.2011.12.001 22198325 PMC3786143

[13] Šmajs D, Strouhal M, Knauf S. Genetics of human and animal uncultivable treponemal pathogens. Infect Genet Evol. 2018; 61:92–107. 10.1016/j.meegid.2018.03.015 29578082

[14] Noordhoek GT, Engelkens HJ, Judanarso J, van der Stek J, Aelbers GN, van der Sluis JJ, et al Yaws in West Sumatra, Indonesia: clinical manifestations, serological findings and characterisation of new *Treponema* isolates by DNA probes. Eur J Clin Microbiol Infect Dis. 1991;10: 12–19. 2009873 10.1007/BF01967091

[15] Weinstock GM, Šmajs D, Hardham J, Norris SJ. From microbial genome sequence to applications. Res Microbiol. 2000;151: 151–158. 10865961 10.1016/s0923-2508(00)00115-7

[16] Bolger AM, Lohse M, Usadel B. Trimmomatic: a flexible trimmer for Illumina sequence data. Bioinformatics. 2014;30: 2114–2120. 10.1093/bioinformatics/btu170 24695404 PMC4103590

[17] Mikalová L, Strouhal M, Čejková D, Zobaníková M, Pospíšilová P, Norris SJ, et al Genome analysis of *Treponema pallidum* subsp. *pallidum* and subsp. *pertenue* strains: most of the genetic differences are localized in six regions. PLoS One. 2010;5: e15713 10.1371/journal.pone.0015713 21209953 PMC3012094

[18] Kearse M, Moir R, Wilson A, Stones-Havas S, Cheung M, Sturrock S, et al Geneious Basic: an integrated and extendable desktop software platform for the organization and analysis of sequence data. Bioinformatics. 2012;28: 1647–1649. 10.1093/bioinformatics/bts199 22543367 PMC3371832

[19] Tamura K, Nei M. Estimation of the number of nucleotide substitutions in the control region of mitochondrial DNA in humans and chimpanzees. Mol Biol Evol. 1993;10: 512–526. 10.1093/oxfordjournals.molbev.a040023 8336541

[20] Kumar S, Stecher G, Tamura K. MEGA7: molecular evolutionary genetics analysis version 7.0 for bigger datasets. Mol Biol Evol. 2016;33: 1870–1874. 10.1093/molbev/msw054 27004904 PMC8210823

[21] Pětrošová H, Pospíšilová P, Strouhal M, Čejková D, Zobaníková M, Mikalová L, et al Resequencing of Treponema pallidum ssp. pallidum strains Nichols and SS14: correction of sequencing errors resulted in increased separation of syphilis treponeme subclusters. PLoS One. 2013;8: e74319 10.1371/journal.pone.0074319 24058545 PMC3769245

[22] Zobaníková M, Mikolka P, Čejková D, Pospíšilová P, Chen L, Strouhal M, et al Complete genome sequence of Treponema pallidum strain DAL-1. Stand Genomic Sci. 2012;7: 12–21. 10.4056/sigs.2615838 23449808 PMC3570794

[23] Pětrošová H, Zobaníková M, Čejková D, Mikalová L, Pospíšilová P, Strouhal M, et al Whole genome sequence of Treponema pallidum ssp. pallidum, strain Mexico A, suggests recombination between yaws and syphilis strains. PLoS Negl Trop Dis. 2012;6: e1832 10.1371/journal.pntd.0001832 23029591 PMC3447947

[24] Giacani L, Jeffrey BM, Molini BJ, Le HT, Lukehart SA, Centurion-Lara A, et al Complete genome sequence and annotation of the *Treponema pallidum* subsp. *pallidum* Chicago strain. J Bacteriol. 2010;192: 2645–2646. 10.1128/JB.00159-10 20348263 PMC2863575

[25] Giacani L, Iverson-Cabral SL, King JC, Molini BJ, Lukehart SA, Centurion-Lara A. Complete genome sequence of the Treponema pallidum subsp. pallidum Sea81-4 strain. Genome Announc. 2014;2: e00333–14. 10.1128/genomeA.00333-14 24744342 PMC3990758

[26] Šmajs D, Zobaníková M, Strouhal M, Čejková D, Dugan-Rocha S, Pospíšilová P, et al Complete genome sequence of Treponema paraluiscuniculi, strain Cuniculi A: the loss of infectivity to humans is associated with genome decay. PLoS One. 2011;6: e20415 10.1371/journal.pone.0020415 21655244 PMC3105029

[27] Marcais G, Kingsford C. A fast, lock-free approach for efficient parallel counting of occurrences of *k*-mers. Bioinformatics. 2011;27: 764–770. 10.1093/bioinformatics/btr011 21217122 PMC3051319

[28] Rice P, Longden I, Bleasby A. EMBOSS: The European Molecular Biology Open Software Suite. Trends Genet. 2000;16: 276–277. 10827456 10.1016/s0168-9525(00)02024-2

[29] Quinlan AR, Hall IM. BEDTools: a flexible suite of utilities for comparing genomic features. Bioinformatics. 2010;26: 841–842. 10.1093/bioinformatics/btq033 20110278 PMC2832824

[30] R Core Team. R: A language and environment for statistical computing. R Foundation for Statistical Computing, Vienna, Austria 2017 Available from: https://www.R-project.org/

[31] Harper KN, Liu H, Ocampo PS, Steiner BM, Martin A, Levert K, et al The sequence of the acidic repeat protein (*arp*) gene differentiates venereal from nonvenereal Treponema pallidum subspecies, and the gene has evolved under strong positive selection in the subspecies that causes syphilis. FEMS Immunol Med Microbiol. 2008;53: 322–332. 10.1111/j.1574-695X.2008.00427.x 18554302

[32] Čejková D, Zobaníková M, Pospíšilová P, Strouhal M, Mikalová L, Weinstock GM, et al Structure of *rrn* operons in pathogenic non-cultivable treponemes: sequence but not genomic position of intergenic spacers correlates with classification of *Treponema pallidum* and *T*. *paraluiscuniculi* strains. J Med Microbiol. 2013;62: 196–207. 10.1099/jmm.0.050658-0 23082031 PMC3755535

[33] Centurion-Lara A, Giacani L, Godornes C, Molini BJ, Brinck Reid T, Lukehart SA. Fine analysis of genetic diversity of the tpr gene family among treponemal species, subspecies and strains. PLoS Negl Trop Dis. 2013;7: e2222 10.1371/journal.pntd.0002222 23696912 PMC3656149

[34] Centurion-Lara A, LaFond RE, Hevner K, Godornes C, Molini BJ, Van Voorhis WC, et al Gene conversion: a mechanism for generation of heterogeneity in the *tprK* gene of *Treponema pallidum* during infection. Mol Microbiol. 2004;52: 1579–1596. 10.1111/j.1365-2958.2004.04086.x 15186410

[35] LaFond RE, Centurion-Lara A, Godornes C, Rompalo AM, Van Voorhis WC, Lukehart SA. Sequence diversity of *Treponema pallidum* subsp. *pallidum tprK* in human syphilis lesions and rabbit-propagated isolates. J Bacteriol. 2003;185: 6262–6268. 10.1128/JB.185.21.6262-6268.2003 14563860 PMC219401

[36] LaFond RE, Centurion-Lara A, Godornes C, Van Voorhis WC, Lukehart SA. *TprK* sequence diversity accumulates during infection of rabbits with *Treponema pallidum* subsp. *pallidum* Nichols strain. Infect Immun. 2006;74: 1896–1906. 10.1128/IAI.74.3.1896-1906.2006 16495565 PMC1418662

[37] Heymans R, Kolader ME, van der Helm JJ, Coutinho RA, Bruisten SM. *TprK* gene regions are not suitable for epidemiological syphilis typing. Eur J Clin Microbiol Infect Dis. 2009;28: 875–878. 10.1007/s10096-009-0717-5 19229562

[38] Čejková D, Strouhal M, Norris SJ, Weinstock GM, Šmajs D. A retrospective study on genetic heterogeneity within Treponema strains: subpopulations are genetically distinct in a limited number of positions. PLoS Negl Trop Dis. 2015;9: e0004110 10.1371/journal.pntd.0004110 26436423 PMC4593590

[39] Giacani L, Lukehart S, Centurion-Lara A. Length of guanosine homopolymeric repeats modulates promoter activity of subfamily II *tpr* genes of *Treponema pallidum* ssp. *pallidum*. FEMS Immunol Med Microbiol. 2007;51: 289–301. 10.1111/j.1574-695X.2007.00303.x 17683506 PMC3006228

[40] Pinto M, Borges V, Antelo M, Pinheiro M, Nunes A, Azevedo J, et al Genome-scale analysis of the non-cultivable Treponema pallidum reveals extensive within-patient genetic variation. Nat Microbiol. 2016;2: 16190 10.1038/nmicrobiol.2016.190 27748767

[41] Radolf JD, Kumar S. The *Treponema pallidum* outer membrane. Curr Top Microbiol Immunol. 2018;415: 1–38. 10.1007/82_2017_44 28849315 PMC5924592

[42] Yang J, Zhang Y. I-TASSER server: new development for protein structure and function predictions. Nucleic Acids Res. 2015;43: W174–W181. 10.1093/nar/gkv342 25883148 PMC4489253

[43] Naqvi AA, Shahbaaz M, Ahmad F, Hassan MI. Identification of functional candidates amongst hypothetical proteins of *Treponema pallidum* ssp. *pallidum*. PLoS One. 2015;10: e0124177 10.1371/journal.pone.0124177 25894582 PMC4403809

[44] Turner TB, Hollander DH. Biology of the treponematoses based on studies carried out at the International Treponematosis Laboratory Center of the Johns Hopkins University under the auspices of the World Health Organization. Monogr Ser World Health Organ. 1957;35: 3–266. 13423342

[45] Chi KH, Danavall D, Taleo F, Pillay A, Ye T, Nachamkin E, et al Molecular differentiation of *Treponema pallidum* subspecies in skin ulceration clinically suspected as yaws in Vanuatu using real-time multiplex PCR and serological methods. Am J Trop Med Hyg. 2015;92: 134–138. 10.4269/ajtmh.14-0459 25404075 PMC4347369

[46] Mikalová L, Šmajs D. Low-dose versus standard-dose azithromycin for treatment of yaws. Lancet Glob Health. 2018; 4: e357–e358. 10.1016/S2214-109X(18)30067-6 29456192

[47] Gray RR, Mulligan CJ, Molini BJ, Sun ES, Giacani L, Godornes C, et al Molecular evolution of the *tprC*, *D*, *I*, *K*, *G*, and *J* genes in the pathogenic genus *Treponema*. Mol Biol Evol. 2006;23: 2220–2233. 10.1093/molbev/msl092 16926243

[48] Godornes C, Giacani L, Barry AE, Mitja O, Lukehart SA. Development of a Multilocus Sequence Typing (MLST) scheme for Treponema pallidum subsp. pertenue: application to yaws in Lihir Island, Papua New Guinea. PLoS Negl Trop Dis. 2017;11: e60006113 10.1371/journal.pntd.0006113 29281641 PMC5760108

[49] Pillay A, Liu H, Chen CY, Holloway B, Sturm AW, Steiner B, et al Molecular subtyping of Treponema pallidum subspecies pallidum. Sex Transm Dis. 1998;25: 408–414. 9773432 10.1097/00007435-199809000-00004

[50] Flasarová M, Šmajs D, Matějková P, Woznicová V, Heroldová-Dvoráková M, Votava M. Molecular detection and subtyping of Treponema pallidum subsp. pallidum in clinical specimens. Epidemiol Mikrobiol Imunol. 2006;55: 105–111. 16970074

[51] Woznicová V, Šmajs D, Wechsler D, Matějková P, Flasarová M. Detection of *Treponema pallidum* subsp. *pallidum* from skin lesions, serum, and cerebrospinal fluid in an infant with congenital syphilis after clindamycin treatment of the mother during pregnancy. J Clin Microbiol. 2007;45: 659–661. 10.1128/JCM.02209-06 17151205 PMC1829021

[52] Flasarová M, Pospíšilová P, Mikalová L, Vališová Z, Dastychová E, Strnadel R, et al Sequencing-based molecular typing of Treponema pallidum strains in the Czech Republic: all identified genotypes are related to the sequence of the SS14 strain. Acta Derm Venereol. 2012;92: 669–674. 10.2340/00015555-1335 22434073

[53] Grillová L, Pĕtrošová H, Mikalová L, Strnadel R, Dastychová E, Kuklová I, et al Molecular typing of *Treponema pallidum* in the Czech Republic during 2011 to 2013: increased prevalence of identified genotypes and of isolates with macrolide resistance. J Clin Microbiol. 2014;52: 3693–3700. 10.1128/JCM.01292-14 25100820 PMC4187743

[54] Mikalová L, Grillová L, Osbak K, Strouhal M, Kenyon C, Crucitti T, et al Molecular typing of syphilis-causing strains among human immunodeficiency virus-positive patients in Antwerp, Belgium. Sex Transm Dis. 2017;44: 376–379. 10.1097/OLQ.0000000000000600 28499290

[55] Gallo Vaulet L, Grillová L, Mikalová L, Casco R, Rodríguez Fermepin M, Pando MA, et al Molecular typing of Treponema pallidum isolates from Buenos Aires, Argentina: frequent Nichols-like isolates and low levels of macrolide resistance. PLoS One. 2017;12: e0172905 10.1371/journal.pone.0172905 28235102 PMC5325558

[56] Brinkman MB, McGill MA, Pettersson J, Rogers A, Matějková P, Šmajs D, et al A novel *Treponema pallidum* antigen, TP0136, is an outer membrane protein that binds human fibronectin. Infect Immun. 2008;76: 1848–1857. 10.1128/IAI.01424-07 18332212 PMC2346692

[57] Ke W, Molini BJ, Lukehart SA, Giacani L. Treponema pallidum subsp. pallidum TP0136 protein is heterogeneous among isolates and binds cellular and plasma fibronectin via its NH2-terminal end. PLoS Negl Trop Dis. 2015;9: e0003662 10.1371/journal.pntd.0003662 25793702 PMC4368718

[58] Giacani L, Brandt SL, Puray-Chavez M, Brinck Reid T, Godornes C, Molini BJ, et al Comparative investigation of the genomic regions involved in antigenic variation of the TprK antigen among treponemal species, subspecies, and strains. J Bacteriol. 2012;194: 4208–4225. 10.1128/JB.00863-12 22661689 PMC3416249

[59] Anand A, Luthra A, Dunham-Ems S, Caimano MJ, Karanian C, LeDoyt M, et al TprC/D (Tp0117/131), a trimeric, pore-forming rare outer membrane protein of Treponema pallidum, has a bipartite domain structure. J Bacteriol. 2012;194: 2321–2333. 10.1128/JB.00101-12 22389487 PMC3347077

[60] Anand A, LeDoyt M, Karanian C, Luthra A, Koszelak-Rosenblum M, Malkowski MG, et al Bipartite topology of *Treponema pallidum* repeat proteins C/D and I: outer membrane insertion, trimerization, and porin function require a C-terminal β-barrel domain. J Biol Chem. 2015;290: 12313–12331. 10.1074/jbc.M114.629188 25805501 PMC4424362

