# Supplementary material for: Complete genome sequences of two strains of Treponema pallidum subsp. pertenue from Indonesia: Modular structure of several treponemal genes
Source: PLoS Negl Trop Dis. 2018 Oct 10;12(10):e0006867. doi: 10.1371/journal.pntd.0006867 (PMC6197692; doi:10.1371/journal.pntd.0006867)
Supplement: S7 Table — (PDF) [file pntd.0006867.s007.pdf]

S7 Table. The overview of predicted structure for module sequences in TP0856 and TP0858 genes

Module sequence in TP0856 and TP0858	Structure prediction according to I-TASSER server*	External localization of coils
**r1**	8 aa** β-strand, 13 aa coil	yes
**r2**	8 aa β-strand, 13 aa coil	yes
**r3**	3 aa coil, 10 aa β-strand	no
**r4**	9 aa coil, 4 aa β-strand, 1 aa coil	yes
**r5**	13 aa coil	yes
**r6**	13 aa coil	yes
**r7**	2 aa coil, 10 aa β-strand	no
**r8**	7 aa coil, 7 aa α-helix, 1 aa coil	yes
**r9**	9 aa coil, 5 aa α-helix	yes

*Yang and Zhang, 2015

**aa, amino acids

References to S7 Table:

Yang J, Zhang Y. I-TASSER server: new development for protein structure and function predictions. Nucleic Acids Res. 2015;43: W174-W181.
