# Supplementary material for: Complete genome sequences of two strains of Treponema pallidum subsp. pertenue from Indonesia: Modular structure of several treponemal genes
Source: PLoS Negl Trop Dis. 2018 Oct 10;12(10):e0006867. doi: 10.1371/journal.pntd.0006867 (PMC6197692; doi:10.1371/journal.pntd.0006867)
Supplement: S1 File — The tree was constructed using the Maximum Likelihood method based on the Tamura-Nei model [19] and MEGA software [20]. The bar scale corresponds to a difference of 0.0001 nucleotides per site. Bootstrap values based on 1,000 replications are shown next to the branches. There were a total of 1,104,654 positions in the final dataset. Both TPE strains of Indonesian origin were highly related to each other when compared to the genetic diversity detected among other, previously characterized TPE strains. TPE strains from Solomon Islands [10] did not show clustering of TPE Kampung Dalan K363 and TPE Sei Geringging K403 with these strains despite their close geographical origin. Draft genome sequences from study by Marks et al. [10] are marked by prefix ERR and can be accessible from GenBank database using the corresponding name e.g. ERR1470334. (PDF) [file pntd.0006867.s008.pdf]

S1 File. A tree constructed from the whole genome sequence alignment of available TPE and TEN genome sequences including also the draft genomes of TPE strains isolated on Solomon Islands [Marks et al., 2017]. The tree was constructed using the Maximum Likelihood method based on the Tamura-Nei model [Tamura and Nei, 1993] and MEGA software [Kumar et al., 2016]. The bar scale corresponds to a difference of 0.0001 nucleotides per site. Bootstrap values based on 1,000 replications are shown next to the branches. There were a total of 1,104,654 positions in the final dataset. Both TPE strains of Indonesian origin were highly related to each other when compared to the genetic diversity detected among other, previously characterized TPE strains. TPE strains from Solomon Islands [Marks et al., 2017] did not show clustering of TPE Kampung Dalan K363 and TPE Sei Geringging K403 with these strains despite their close geographical origin. Draft genome sequences from study by Marks *et al.* (2017) are marked by prefix ERR and can be accessible from GenBank database using the corresponding name e.g. ERR1470334.

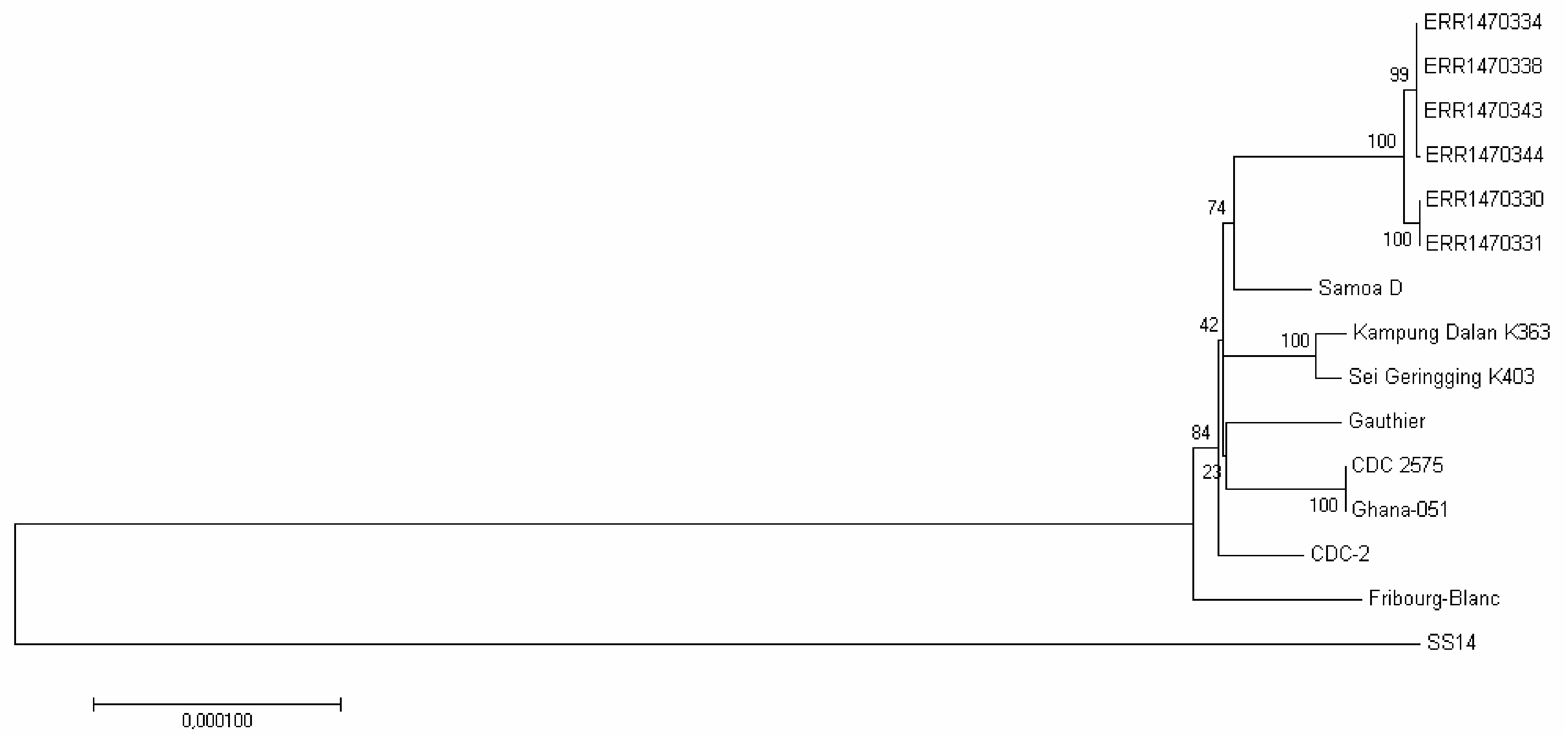

References to S1 file:

Marks M, Fookes M, Wagner J, Butcher R, Ghinai R, Sokana O, et al. Diagnostics for yaws eradication: insights from direct next generation sequencing of cutaneous strains of *Treponema pallidum*. Clin Infect Dis. 2017 Oct 16. doi: 10.1093/cid/cix892.

Tamura K, Nei M. Estimation of the number of nucleotide substitutions in the control region of mitochondrial DNA in humans and chimpanzees. Mol Biol Evol. 1993;10: 512-526.

Kumar S, Stecher G, Tamura K. MEGA7: molecular evolutionary genetics analysis version 7.0 for bigger datasets. Mol Biol Evol. 2016;33: 1870-1874. doi: 10.1093/molbev/msw054.
